# Observations on the Cause, Nature, and Treatment, Etc.

**Published:** 1854-06

**Authors:** 


### BOOK NOTICES.

*Observations on the Cause, Nature, and Treatment of Epidemic Cholera.* By A. B. PALMER, M. D., and Professor of Anatomy in the University of Michigan. From the Author.

THIS is a monograph of 37 pages, made up of extra sheets from the *Peninsular Journal of Medicine*, in which the matter originally appeared. The author discusses the general subject under three heads, viz:—the causes, essential and predisposing—the symptomatology and pathology—and the treatment.

Under the first head he gives a cursory, but interesting review of the various theories and speculations which have been advanced by writers on this subject.

Several pages are devoted to a consideration of these hypotheses, the writer apparently satisfying himself fully that the cholera depends directly upon the existence and diffusion of a specific poison, *sui generis*, and everywhere identical. Whether such poison belongs to the vegetable, animal, or inorganic world, he does not inform us, but thinks its existence and action can be explained equally well on either of the general hypotheses which have been broached by eminent writers.

We have at present neither time nor space to enter upon a critical examination of this supposed specific cholera poison. We however, believe *it* to be purely hypothetical, and the circumstances alledged as proof of its existence, for the most part, naked assumptions, as we shall on some future occasion attempt to show more at length. That part of the monograph relating to the predisposing causes of cholera is much more interesting and valuable. We fully agree with the following paragraph:

"But we proceed to a branch of the subject less doubtful in its nature, and more practical in its character; viz: The *predisposing cause* of cholera,—or the circumstances and conditions, as it regards localities and persons, which favor the spread of the essential cholera-cause, and render it more effectual and fatal. Whatever view may be taken respecting the nature, or even the existence of the specific poison it is certain that in order to give it [illegible] potency and effect certain other conditions must exist These have been much dwelt upon, and their general features pretty well established in the opinion of the profession It should be confessed, however, that in particular cases, general rules have been set at defiance.—the facts, however, are sufficiently uniform to establish the general rules Briefly and generally stated, the localities most liable to its spread, other things being equal, are low moist, and particularly filthy situations Warm climates and seasons rather more favorable than cold, and a densely populated region more then one scarcely settled. It is more liable to follow water courses and thoroughfares. partly because these are usually more low filthy. and densely populated and partly. we believe because the morbid matter is conveyed by intercommunication Still it takes general and sometimes particular causes in obedience to laws we do not understand In regard to classes of persons most liable to be attacked and to become its victims—the intemperate, the destitute. the filthy. the vicious. the enfeebled, the terrified, and the degraded, are immeasurably more subject to its ravages than those in opposite conditions"

As City Physician during one season, when the cholera prevailed to some extent in the city, Dr. Palmer had an excellent opportunity to observe the different degrees to which different classes of people, affected by different habits and circumstances, became subject to the disease And we are happy to find that his observations fully coincide with our own in relation to these points In reference to the influence of intoxicating or alcoholic drinks, the Doctor makes the following judicious remarks which we particularly commend to the notice of profession

"It may be alleged that the exciting and stimulating effects of these drinks in moderate quantities, may enable the system more effectually to resist a morbid influence, and this might be true if the only effect of alcohol was stimulating and further, if that stimulation could constantly be kept up at a uniform and proper point; but alcohol has other effects than stimulation, and as we have seen, of a deleterious character, and that stimulation cannot be kept uniform. By the fixed laws of the vital economy all unnatural excitement is followed by a corresponding degree of depression, and the whole vital condition and movements of the spirit consumer are ever fluctuating and unsteady. If then, the excitement would resist the morbid influence, the subsequent depression would favor the productiou of its effects. Both reason and experience show that the belief in the preventive virtues of alcohol in this disease, is fallacious, and affords an example of those popular delusions which our enlightened profession should labor to destroy."

The directions given in reference to the treatment of the cholera, both prophylactic and curative, are in the main judicious and proper. Though the pamphlet before us contains little or nothing that is new on the subject of which it treats. yet it contains some facts of value, together with the practical experience of a reflecting and judicious physician, and is hence well worthy of perusal by the profession generally. D.

